# Supplementary material for: Characterizing Molecular and Synaptic Signatures in mouse models of Late-Onset Alzheimer’s Disease Independent of Amyloid and Tau Pathology
Source: bioRxiv. 2023 Dec 20:2023.12.19.571985. Preprint. [Version 1] doi: 10.1101/2023.12.19.571985 (PMC10769232; doi:10.1101/2023.12.19.571985)

bioRxiv preprint doi: https://doi.org/10.1101/2023.12.19.571985; this version posted December 20, 2023. The copyright holder for this preprint (which was not certified by peer review) is the author/funder, who has granted bioRxiv a license to display the preprint in perpetuity. It is made available under aCC-BY 4.0 International license.

Supplemental Methods

Cohort 1 – The Jackson Laboratory

Breeding and Husbandry

All mice were housed 5 per cage with SaniChip bedding and initially provided LabDiet® 5K52/5K67 (6% fat; control diet, CD). The mice were kept on a 12-hour light/dark schedule with the lights on from 7:00 a.m. to 7:00 p.m. daily. The animals were ear-punched for identification purposes and subsequently microchipped at the tail base using a p-chip system (PharmaSeq).

Behavioral testing

Behavioral tests were conducted as previously reported [15] in the following order with at minimum a 1-2-day rest period between tests: Frailty assessment with core body temperature recording, open field test, spontaneous alternation, rotarod, and wheel running activity. On each test day, subjects were transported from the adjacent housing room into the procedure room, tails were labeled with a non-toxic permanent marker with the assigned subject ID number, and subjects were left to acclimate undisturbed to the testing environment for a minimum 60 minutes prior to testing. Between subjects, all testing arenas were sanitized with 70% ethanol solution and dried prior to introducing the next subject. Lighting in the testing rooms were consistent with the housing room (~500 lux) unless where specifically noted. At minimum 5 days post the conclusion of behavioral testing, mice were sent for tissue harvesting.

Frailty assessment: Similar to as previously described[15], subjects were individually evaluated for the absence or presence of 26 aging-related characteristic traits and scored a 0, 0.5,

bioRxiv preprint doi: https://doi.org/10.1101/2023.12.19.571985; this version posted December 20, 2023. The copyright holder for this preprint (which was not certified by peer review) is the author/funder, who has granted bioRxiv a license to display the preprint in perpetuity. It is made available under aCC-BY 4.0 International license.

or 1 (based on presence/absence, and severity) for each assessment by a trained observer, blind to genotype/age, and included the following assessments: alopecia; loss of fur color; dermatitis/skin lesions; loss of whiskers; coat condition; piloerection; cataracts; eye discharge/swelling; microphthalmia; nasal discharge; rectal prolapse; vaginal/uterine/penile; diarrhea; vestibular disturbance; vision loss assessed by visual placing upon subject being lowered to a grid; menace reflex; tail stiffening; impaired gait during free walking; tremor; tumors; distended abdomen; kyphosis; body condition; breathing rate/depth; malocclusions; righting reflex. The frailty index score was calculated as the cumulative score of all measures with a maximum score of 26.

Core body temperature: Core body temperature was recorded just prior to the conclusion of the frailty assessment via a glycerol-lubricated thermistor rectal probe (Braintree Scientific product# RET 3; measuring 3/4" L .028 dia. .065 tip) inserted ~2cm into the rectum of a manually restrained mouse for approximately 10 seconds. Temperature was recorded to the nearest 0.1°C (Braintree Scientific product#TH5 Thermalert digital thermometer).

Open field activity: Versamax Open Field Arenas (40cm x 40cm x 40cm; Omnitech Electronics, OH USA) were used for this test. Arenas were housed within sound attenuated chambers with lighting in the testing room and arenas consistent with the housing room (~500 lux). Mice were placed individually into the center of the arena and infrared beams recorded distance traveled (cm), vertical activity, and perimeter/center time. Data were collected in 5-minute time-bins for duration of 60 minutes.

Spontaneous alternation: Mice were acclimated to the testing room under ambient lighting conditions (~ 50 lux). A clear polycarbonate y-maze (in-house fabricated; arm dimensions 33.65cm length, 6cm width, 15cm height) placed on top of an infrared reflecting

bioRxiv preprint doi: https://doi.org/10.1101/2023.12.19.571985; this version posted December 20, 2023. The copyright holder for this preprint (which was not certified by peer review) is the author/funder, who has granted bioRxiv a license to display the preprint in perpetuity. It is made available under aCC-BY 4.0 International license.

background (Noldus, The Netherlands), surrounded by a black floor-to ceiling curtain to minimize extramaze visual cues was used for this test. Mice were placed midway of the start arm, facing the center of the y for an 8-minute test period and the sequence of entries into each arm are recorded via a ceiling-mounted infrared camera integrated with behavioral tracking software (Noldus Ethovision XT). Percent spontaneous alternation is calculated as the number of triads (entries into each of the three different arms of the maze in a sequence of three without returning to a previously visited arm) relative to the number of alteration opportunities.

Rotarod test for motor coordination: An accelerating Rotarod (Ugo-Basile; model 47600) is used for this test. Lighting in the testing room is consistent with the housing room (~ 500 lux). The trial began with mice being placed on the rotating rod (4 rpm), which accelerates up to 40 rpm over the course of 300 seconds. Each mouse is subjected to 3 consecutive trials with an ~1 min inter-trial interval to allow cleaning of the rod between trials. Latency to fall (sec) is measured. Subjects that fall upon initial placement on the rod, before acceleration begins, are scored as 0 sec for that trial.

Wheel running activity: Subjects were individually housed into a clean cage with a running wheel (Med-Associates, Vermont, USA) and with food and water ad libitum. The light cycle was identical to the housing room with 12:12 L:D (lights on at 6:00am). Running Wheels were equipped with a wireless transponder that recorded activity on the running wheels (revolutions) in sync with a computer that time stamps events. Mice were left undisturbed throughout the testing period with the exception of daily welfare checks. Data were evaluated for time spent running (min), total distance traveled (meters), and speed (revolutions per min) over the course of three 24-hour periods.

bioRxiv preprint doi: https://doi.org/10.1101/2023.12.19.571985; this version posted December 20, 2023. The copyright holder for this preprint (which was not certified by peer review) is the author/funder, who has granted bioRxiv a license to display the preprint in perpetuity. It is made available under aCC-BY 4.0 International license.

Behavioral data analysis: Prior to data analysis and while still blinded, results were adjusted to exclude data only from mice which could not be tested or which data was not available inclusive of any equipment failures, escape episodes, etc. Subjects were not excluded by any mathematical determination. Data was analyzed under coded genotypes (A, B, C, etc.) within sex, as one-way or two-way ANOVA as appropriate versus sex- and age- matched WT control. The blind was revealed at the conclusion of the data analysis for interpretation.

Fasted blood glucose collection and measurement

Fasted mice were placed into a fresh cage, free of food but with fresh water, at 6am – the beginning of the light-ON cycle. Mice were fasted for 6 hours, until 12pm, at which time blood glucose levels were analyzed. Prior to mouse restraint, a Contour Next EZ blood glucose monitor (Ascensia, Parsippany, NJ) was calibrated with Contour glucose control solution and Contour Next test strips. While restraining the animal, with a 5.0mm lancet a stab incision was made into and perpendicular to the cheek, located dorsal to the to the cheek skin gland at a distance equal to the height of the eye and caudal distance equal to the length of the eye. One drop of blood, approximately 10µl, was applied to a blood glucose test strip and readings were recorded.

Animal anesthesia

Upon arrival at the terminal endpoint for each aged mouse cohort (4, 12, 18 or 24 months), individual animals were weighed prior to intraperitoneal administration of tribromoethanol (1mg/kg). Routine confirmation of deep anesthesia was performed every 5 minutes by toe pinch.

bioRxiv preprint doi: https://doi.org/10.1101/2023.12.19.571985; this version posted December 20, 2023. The copyright holder for this preprint (which was not certified by peer review) is the author/funder, who has granted bioRxiv a license to display the preprint in perpetuity. It is made available under aCC-BY 4.0 International license.

Whole Animal Perfusion

First confirming deep anesthetization via toe pinch, an incision is made along the ventral midline to expose the thorax and abdomen, followed by removal of the lateral borders of the diaphragm and ribcage revealed the heart. Prior to perfusion trunk blood and CSF samples were collected. To perfuse the animal, a small cut was placed in the right atrium to relieve pressure from the vascular system before perfusing the animal transcardially with 1XPBS via injection into the left ventricle. Completion of perfusion and clearance of the vascular system was indicated by a blanching of the liver. At this time organs of interest were collected as indicated.

Non-fasted blood collection and analysis

Blood was collected by cardiac puncture from non-fasted, anesthetized animals (see Perfusion method) at harvest prior to incision of the right atrium and subsequent perfusion. A 25-gauge EDTA-coated needle, attached to a 1mL syringe, is inserted into the right atrium of the exposed heart and the plunger gently pulled to slowly aspirate approximately 500mL of blood, avoiding entrapping air in the syringe to prevent hemolysis. After removal of the needle from the syringe, the blood was slowly injected into a 1.5mL EDTA coated MAP-K2 blood microtainer (363706, BD, San Jose, CA) on ice. Blood tubes were spun at 4°C and 4,388xg for 15 minutes. Blood serum is then removed and aliquoted equally into three replicate 1.5mL tubes on ice. Tubes were then snap frozen on dry ice and stored long-term at -80°C. Thawed blood plasma collected from non-fasted mice was then analyzed by Beckman Coulter AU680 chemistry analyzer (Beckman Coulter, Brea, CA) and Siemens Advia 120 (Germany) for levels of non-fasted glucose, total

bioRxiv preprint doi: https://doi.org/10.1101/2023.12.19.571985; this version posted December 20, 2023. The copyright holder for this preprint (which was not certified by peer review) is the author/funder, who has granted bioRxiv a license to display the preprint in perpetuity. It is made available under aCC-BY 4.0 International license.

cholesterol, LDL (low-density lipoproteins), HDL (high-density lipoproteins), triglycerides, and NEFA (non-essential fatty acids).

Brain harvest

Anesthetized and subsequently perfused animals were decapitated, and heads submerged quickly in cold 1xPBS. The brain was carefully removed from the skull, weighed, and divided midsagitally, into left and right hemispheres, using a brain matrix. The right hemisphere was quickly homogenized on ice and equally aliquoted into three cryotubes for metabolomic, proteomic, and transcriptomic analysis. Cryotubes were immediately snap frozen on dry ice, and stored long-term at -80°C. The left hemisphere was immediately placed in 5mL 4% PFA at 4°C for no less than 24 hours, but no longer than 30 hours. The left hemisphere was then moved from PFA solution to 10mL 15% sucrose at 4°C for 24 hours, or until it sinks in the sucrose, when it was then transferred to a 30% sucrose for 24 hours at 4°C, or until it sinks in the solution. The left hemisphere was then removed from 30% sucrose solution, snap frozen on a flat mold, cut-side down, floating in 2-methylbutane solution cooled by dry ice. Once frozen the left hemisphere is then placed into a cryotube and stored at -80°C until used for microtome sectioning and immunohistochemistry analysis.

Immunohistochemistry and microscopy imaging

During harvest, whole mouse brains were removed and weighed. Using a brain matrix, left and right hemispheres were separated along the midsagittal plane. The left hemisphere was placed in 5mL of 4% PFA at 4°C overnight, then moved to 10mL of 15% sucrose at 4°C overnight, before

bioRxiv preprint doi: https://doi.org/10.1101/2023.12.19.571985; this version posted December 20, 2023. The copyright holder for this preprint (which was not certified by peer review) is the author/funder, who has granted bioRxiv a license to display the preprint in perpetuity. It is made available under aCC-BY 4.0 International license.

finally being incubated in 10mL of 30% sucrose at 4°C overnight or until brain sinks to bottom of the tube. The left hemisphere was then snap frozen and stored at -80°C until sectioned. Left hemispheres (see preparation in Brain harvest method) were cut via Thermo Scientific HM430 sliding microtome at 25µm thickness. Coronal brain tissue sections were oriented to capture the cortex and hippocampus at approximately Bregma: -2.75mm and Interaural 1.05mm. Each section was placed into cryoprotectant buffer (37.5% 1xPBS, 31.25% glycerol, 31.25% ethylene glycol) for immediate use or long-term storage at -20°C. Floating sections were then blocked prior to immunohistochemical staining and mounting. After blocking slides with 10% normal donkey serum or normal goat serum diluted in 1xPBS+0.5%Triton wash buffer all antibodies were washed floating in 1xPBT (1x PBS with 0.5% Triton) wash buffer after blocking for 1 hour at room temperature on shaker in 10% NGS (normal goat serum) or 10% NDS (normal donkey serum) in 1xPBT. Secondary antibodies were incubated in 10% NGS or NDS in 1xPBT for 1 hour at room temperature, followed by washes in 1xPBT before mounting on to slides. Slides were then imaged on a Leica Versa slide scanner, automated fluorescent microscope system (Leica, Allendale, NJ). For further analysis, regions of the cortex and hippocampus were processed using Imaris (Bitplane, Concord, MA) software to quantify cell counts, fluorescence intensity, and surface area ratios. [Iba1 (Wako, 019-19741, 1:300), DAPI (1:1000), GFAP (Origene, AP31806PU-N, 1:1000), NeuN (abcam, ab104225, 1:500), and ThioS (1% in 50% ethanol)]

Transcriptomics and Proteomics

bioRxiv preprint doi: https://doi.org/10.1101/2023.12.19.571985; this version posted December 20, 2023. The copyright holder for this preprint (which was not certified by peer review) is the author/funder, who has granted bioRxiv a license to display the preprint in perpetuity. It is made available under aCC-BY 4.0 International license.

RNA-Seq and Tandem Mass Tag Proteomics data were obtained from whole left hemisphere brain samples from mice expressing human *APOEε4* and the *Trem2*R47H* mutation (*APOEε4.Trem2*R47H*; strain name LOAD1) [8] and mice expressing humanized Aβ in combination with these genetic risk factors (*APOEε4.Trem2*R47H*.hAβ; strain name LOAD2). Whole-brain left hemispheres were collected at 4, 12, 18, and 24 months of age from both sexes and both diets (standard chow and high-fat, high-sugar diet) where available. Additional C57BL/6J (B6) mice at 18 months were included as a standard aged control. At least six biological replicates were collected for each sample group.

RNA-Sequencing data analysis

RNA-Seq data were processed using nf-core/rnaseq pipeline [https://doi.org/10.5281/zenodo.1400710]. Briefly, reads were aligned to the reference mouse genome (version GRCm38.p6) using STAR [41] and gene expression was quantified with RSEM [42]. To measure human *APOE* gene expression, we created a custom mouse reference genome by concatenating human *APOE* gene sequence (human chromosome 19:44905754-44909393; build GRCh38.p10) into the mouse genome (GRCm38.p6) as a separate chromosome (referred as chromosome 21 in chimeric mouse genome). Subsequently, we added a gene annotation for the human *APOE* gene into the mouse gene annotation file.

Differential gene and protein expression analysis

Differentially expressed genes in mouse models were identified using the R Bioconductor package DESeq2 (v1.16.1) [43]. We used the Benjamini-Hochberg corrected p-values with a significance

bioRxiv preprint doi: https://doi.org/10.1101/2023.12.19.571985; this version posted December 20, 2023. The copyright holder for this preprint (which was not certified by peer review) is the author/funder, who has granted bioRxiv a license to display the preprint in perpetuity. It is made available under aCC-BY 4.0 International license.

threshold of 0.05 to identify differentially expressed genes. We performed differential protein expression analysis for each mouse model compared to age and sex-matched control mice using one-way ANOVA followed by post hoc correction using Tukey HSD test to match methods used in analogous human studies [19].

Functional enrichment analysis

Functional annotations and enrichment analyses were performed using the R Bioconductor package clusterProfiler [44], with Gene Ontology term and KEGG pathway enrichment analyses performed using functions enrichGO and enrichKEGG, respectively. The function compareCluster was used to compare enriched functional categories of each gene module. The significance threshold for all enrichment analyses was set to 0.05 using Benjamini-Hochberg adjusted p-values.

Weighted gene co-expression network analysis of the mouse transcriptome

Weighted gene co-expression network analysis (WGCNA) [45] was performed to identify modules (clusters) of correlated genes using log TPM normalized expression values. We used the step-by-step construction approach for network construction and module identification [17]. The default unsigned network type was used, and a soft thresholding power of 5 was chosen to meet the scale-free topology criterion in the pickSoftThreshold function. We set the minimum modules size as 30, and merged modules whose correlation coefficient were greater than 0.75 (mergeCutHeight = 0.25). Each module is summarized by the module eigengene (ME), defined as first principal component of the gene expression profiles of each module. Further we computed the Pearson

bioRxiv preprint doi: https://doi.org/10.1101/2023.12.19.571985; this version posted December 20, 2023. The copyright holder for this preprint (which was not certified by peer review) is the author/funder, who has granted bioRxiv a license to display the preprint in perpetuity. It is made available under aCC-BY 4.0 International license.

correlation coefficient of modules to genotype, age, sex, behavioral data, cytokines levels, and cell count data.

Enrichment of AD biological domains

Gene functional enrichment analyses are informative but sometimes it is difficult to understand how the enriched terms relate to the biology of AD. Cary et al. [18] developed 19 biological domains that capture the AD-associated endophenotypes and defined them using an exhaustive set of Gene Ontology (GO) terms, with the intent to keep each domain siloed in a biologically coherent fashion. We performed Fisher's exact tests to identify enrichment of each AD biological domain and resident GO terms in WGCNA module gene sets.

Linear regression Analyses

Multiple behavioral data, cytokines levels, NfL, and cell counts were measured for each animal for multiple ages from both sexes. We performed multiple linear regression model to determine the effect of each variant (age, sex, high-fat diet, and genotypes) on these phenotypic data:

$$value \sim \beta_0 + \beta_{age} + \beta_{sex} + \beta_{LOAD1.CD} + \beta_{LOAD2.CD} + \beta_{LOAD1.HFD} + \beta_{LOAD2.HFD}$$

Here the value represents measurements of each quantitative phenotype of interest. CD and HFD refer to chow and high-fat diets, respectively, to quantify the effects of genotypes paired with diet.

Proteomics experimental design and data processing

bioRxiv preprint doi: https://doi.org/10.1101/2023.12.19.571985; this version posted December 20, 2023. The copyright holder for this preprint (which was not certified by peer review) is the author/funder, who has granted bioRxiv a license to display the preprint in perpetuity. It is made available under aCC-BY 4.0 International license.

Tissue Homogenization and Protein Digestion

Samples were homogenized in 8 M urea lysis buffer (8 M urea, 10 mM Tris, 100 mM NaH2PO4, pH 8.5) with HALT protease and phosphatase inhibitor cocktail (ThermoFisher) using a Bullet Blender (NextAdvance) essentially described [46]. Each Rino sample tube (NextAdvance) was supplemented with ~100 µL of stainless-steel beads (0.9 to 2.0 mm blend, NextAdvance) and 300 µL of lysis buffer. Tissues were added immediately after excision and homogenized with bullet blender at 4 °C with 2 full 5 min cycles. The lysates were transferred to new Eppendorf Lobind tubes and sonicated for 3 cycles consisting of 5 s of active sonication at 30% amplitude, followed by 15 s on ice. Samples were then centrifuged for 5 min at 15,000 x g and the supernatant transferred to a new tube. Protein concentration was determined by bicinchoninic acid (BCA) assay (Pierce). For protein digestion, 100 µg of each sample was aliquoted and volumes normalized with additional lysis buffer. Samples were reduced with 5 mM dithiothreitol (DTT) at room temperature for 30 min, followed by 10 mM iodoacetamide (IAA) alkylation in the dark for another 30 min. Lysyl endopeptidase (Wako) at 1:25 (w/w) was added, and digestion allowed to proceed overnight. Samples were then 7-fold diluted with 50 mM ammonium bicarbonate. Trypsin (Promega) was then added at 1:25 (w/w) and digestion proceeded overnight. The peptide solutions were acidified to a final concentration of 1% (vol/vol) formic acid (FA) and 0.1% (vol/vol) trifluoroacetic acid (TFA) and desalted with a 30 mg HLB column (Oasis). Each HLB column was first rinsed with 1 mL of methanol, washed with 1 mL 50% (vol/vol) acetonitrile (ACN), and equilibrated with 2×1 mL 0.1% (vol/vol) TFA. The samples were then loaded onto the column and washed with 2×1 mL 0.1% (vol/vol) TFA. Elution was performed with 2 volumes of 0.5 mL 50% (vol/vol) ACN.

bioRxiv preprint doi: https://doi.org/10.1101/2023.12.19.571985; this version posted December 20, 2023. The copyright holder for this preprint (which was not certified by peer review) is the author/funder, who has granted bioRxiv a license to display the preprint in perpetuity. It is made available under aCC-BY 4.0 International license.

Isobaric Tandem Mass Tag (TMT) Peptide Labeling

Each sample (containing 100 µg of peptides) was re-suspended in 100 mM TEAB buffer (100 µL). The TMT labeling reagents (5mg) were equilibrated to room temperature, and anhydrous ACN (256 µL) was added to each reagent channel. Each channel was gently vortexed for 5 min, and then 41 µL from each TMT channel was transferred to the peptide solutions and allowed to incubate for 1 h at room temperature. The reaction was quenched with 5% (vol/vol) hydroxylamine (8 µl) (Pierce). All channels were then combined and dried by SpeedVac (LabConco) to approximately 150 µL and diluted with 1 mL of 0.1% (vol/vol) TFA, then acidified to a final concentration of 1% (vol/vol) FA and 0.1% (vol/vol) TFA. Labeled peptides were desalted with a 200 mg C18 Sep-Pak column (Waters). Each Sep-Pak column was activated with 3 mL of methanol, washed with 3 mL of 50% (vol/vol) ACN, and equilibrated with 2×3 mL of 0.1% TFA. The samples were then loaded and each column was washed with 2×3 mL 0.1% (vol/vol) TFA, followed by 2 mL of 1% (vol/vol) FA. Elution was performed with 2 volumes of 1.5 mL 50% (vol/vol) ACN. The eluates were then dried to completeness using a SpeedVac.

High-pH Off-line Fractionation

High pH off line fractionation was conducted essentially essentially described [46]. Dried samples were re-suspended in high pH loading buffer (0.07% vol/vol NH4OH, 0.045% vol/vol FA, 2% vol/vol ACN) and loaded onto a Water's BEH 1.7 um 2.1mm by 150mm. An Thermo Vanquish was used to carry out the fractionation. Solvent A consisted of 0.0175% (vol/vol) NH4OH, 0.01125% (vol/vol) FA, and 2% (vol/vol) ACN; solvent B consisted of 0.0175% (vol/vol) NH4OH, 0.01125% (vol/vol) FA, and 90% (vol/vol) ACN. The sample elution was performed over a 25 min gradient with a flow rate of 0.6 mL/min. A total of 192 individual equal volume

bioRxiv preprint doi: https://doi.org/10.1101/2023.12.19.571985; this version posted December 20, 2023. The copyright holder for this preprint (which was not certified by peer review) is the author/funder, who has granted bioRxiv a license to display the preprint in perpetuity. It is made available under aCC-BY 4.0 International license.

fractions were collected across the gradient and subsequently pooled by concatenation into 96 fractions and dried to completeness using a SpeedVac.

LC-MS/MS Methods

All fractions were resuspended in an equal volume of loading buffer (0.1% FA, 0.03% TFA, 1% ACN) and analyzed by liquid chromatography coupled to tandem mass spectrometry. Peptide eluents were separated on a custom in-house packed CSH 1.7um (15 cm × 150 µM internal diameter (ID) by a Dionex RSLCnano UPLC (ThermoFisher Scientific). Buffer A was water with 0.1% (vol/vol) formic acid, and buffer B was 80% (vol/vol) acetonitrile in water with 0.1% (vol/vol) formic acid. Elution was performed over a 32 min gradient with flow rate at 1000 nL/min. The gradient was from 1% to 99% solvent B. Peptides were monitored on a Orbitrap Eclipse mass spectrometer with a high-field asymmetric waveform ion mobility spectrometry (FAIMS Pro) ion mobility source(ThermoFisher Scientific). Two compensation voltages (CV) were chosen for the FAIMS. For each CV (-45 and -65) top speed cycle of 1.5 seconds, the full scan (MS1) was performed with an m/z range of 410-1600 at 60,000 resolution at standard settings. The higher energy collision-induced dissociation (HCD) tandem scans were collected at 35% collision energy with an isolation of 0.7 m/z, a resolution of 30,000 with TurboTMT on, an AGC setting of 250% normalized agc target, and a maximum injection time of 54 ms. Dynamic exclusion was set to exclude previously sequenced peaks for 15 seconds within a 10-ppm isolation window.

Database Search

bioRxiv preprint doi: https://doi.org/10.1101/2023.12.19.571985; this version posted December 20, 2023. The copyright holder for this preprint (which was not certified by peer review) is the author/funder, who has granted bioRxiv a license to display the preprint in perpetuity. It is made available under aCC-BY 4.0 International license.

Datasets (672 raw files (n=7 batches of 96 high pH fractions)) were searched using FragPipe (version 20.0). The FragPipe pipeline relies on MSFragger (version 3.8; [47, 48]) for peptide identification and Philosopher (version 5.0.0; [49]) for FDR filtering and downstream processing. The mouse protein database used contains canonical isoforms from Uniprot/Swissprot as of 02/2023. The workflow used in FragPipe followed default TMT-16 plex parameters, used for both TMT-16 and TMT-18 experimental design. Briefly, precursor mass tolerance was -20 to 20 ppm, fragment mass tolerance of 20 ppm, mass calibration and parameter optimization were selected, and isotope error was set to -1/0/1/2/3. Enzyme specificity was set to strict-trypsin and up to two missing trypsin cleavages were allowed. Peptide length was allowed to range from 7 to 50 and peptide mass from either 200 to 5,000 Da. Variable modifications that were allowed in our search included: oxidation on methionine, N-terminal acetylation on protein, and N-terminal acetylation on peptide, with a maximum of 3 variable modifications per peptide. Peptide Spectral Matches were validated using Percolator[50]. The false discovery rate (FDR) threshold was set to 1% and protein and peptide abundances were quantified using Philosopher for downstream analysis.

Protein Quantitation and Normalization

The protein abundances are normalized by scaling total protein signal within each channel for each specific case sample to the maximum channel-specific total signal. We then used a tunable median polish approach, TAMPOR, to remove technical batch variance in the proteomic data, as previously described[51]. TAMPOR is utilized to remove intra-batch and inter-batch variance while preserving meaningful biological variance in protein abundance values, normalizing to the median of selected intra-batch samples. This approach is robust to outliers and columns with up to 50% values missing. If a protein had more than 50% samples with missing values, it was removed from

bioRxiv preprint doi: https://doi.org/10.1101/2023.12.19.571985; this version posted December 20, 2023. The copyright holder for this preprint (which was not certified by peer review) is the author/funder, who has granted bioRxiv a license to display the preprint in perpetuity. It is made available under aCC-BY 4.0 International license.

the matrix. No imputation of missing values was performed for any cohort. For the current data, TAMPOR leverages the median protein abundance from the pooled Global Internal Standard (GIS) TMT channels as the denominators in both factors to normalize sample-specific protein abundances across the 7 batches of samples.

AD-related protein co-expression modules from human postmortem brain tissue

Human protein co-expression modules were obtained from previously published protein expression profiles from human dorsolateral prefrontal frontal cortex [19]. Briefly, Johnson et al. [19] analyzed more than 500 dorsal prefrontal cortex (DLPFC) tissues from control, asymptomatic AD (AsymAD), and AD brains across multiple centers and cohorts using tandem mass tag mass spectrometry (TMT-MS) based quantitative proteomics and generated a deep TMT AD protein network using WGCNA. This network consists of 44 modules of proteins related to one another by their co-expression across control and disease tissues. Johnson, et al. functionally annotated these modules using Gene Ontology analysis of its constituent proteins and assigned cell types to each module, and measured correlations of each summary module eigenprotein to neuropathological or cognitive traits present in the cohorts [19]. We obtained case-control LogFC values previously adjusted for sex for each quantified protein and membership in protein co-expression modules from the AD Knowledge Portal (https://www.synapse.org/#!Synapse:syn25453861).

Mouse-human correlation analysis

bioRxiv preprint doi: https://doi.org/10.1101/2023.12.19.571985; this version posted December 20, 2023. The copyright holder for this preprint (which was not certified by peer review) is the author/funder, who has granted bioRxiv a license to display the preprint in perpetuity. It is made available under aCC-BY 4.0 International license.

To compare mouse expression changes with those observed in human disease, we computed Pearson correlations between changes in expression (log2 fold change) in human AD cases versus controls and the changes in expression (log2 fold change) in each mouse model contributed by a genotype-diet combination from the linear model (*i.e.* corrected for sex analogous to the human data). Correlations were computed across the set of orthologous proteins in each protein module [52, 53] using cor.test function in R as:

$$\text{cor.test}\ (\text{LogFC(h)}, \text{LogFC(m)}) \qquad (1)$$

where LogFC(h) is a vector of protein log folds changes in human AD cases compared to controls and LogFC(m) is a vector of protein log fold changes in LOAD mouse models from the linear model.

Cohort 2 – Indiana University

In vivo MR imaging

LOAD2 mice were imaged at the age of 4 months, 12 months and 18 months respectively. For male mice, there were 9 control (CON) and 7 high fat (HF) at 4 months, 9 CON and 10 HF at 12 months, and 7 CON and 10 HF at 18 months respectively. For female mice, there were 10 CON and 9 HF at 4 months, 10 CON and 8 HF, and 8 CON and 4 HF at 18 months respectively.

MR images of the specimens were acquired on a 30-cm bore 9.4 Tesla magnet (Bruker BioSpec 94/30, Billerica, MA, United States) with a maximum gradient strength of 660 mT/m on each axis. A high-sensitivity cryogenic RF surface receive-only coil was used for signal reception (Bruker Cryoprobe). A multi-echo two-dimensional Rapid Acquisition with Relaxation

bioRxiv preprint doi: https://doi.org/10.1101/2023.12.19.571985; this version posted December 20, 2023. The copyright holder for this preprint (which was not certified by peer review) is the author/funder, who has granted bioRxiv a license to display the preprint in perpetuity. It is made available under aCC-BY 4.0 International license.

Enhancement (RARE) pulse sequence with 8 echo train length (ETL) was used to acquire T2-weighted images. The scanning parameters were as follows: matrix size = 304 x 256, field-of-view (FOV) = 15.2 x 12.8 mm^2, in-plane resolution = 100 µm x 100 µm, slice thickness = 250 µm, and repetition time (TR) = 7 s.

MRI Data analysis: All Digital Imaging and Communications in Medicine (DICOM) raw data were converted into Neuroimaging Informatics Technology Initiative (NIfTI) format. The mask was created using U-Net[54]. The data were masked and registered to standard T2W template using non-linear diffeomorphic ANTs registration[55]. The template brain labels were warped into subject space following the registration. The mean volume of each label and whole brain volume were calculated, and t-test statistics were conducted to determine statistical significance. The p-value was set at 0.05 level of significance.

Perfusion and Preparation of Blood and Tissue Samples

Mice at Indiana University were anesthetized to the surgical plane of anesthesia with tribromoethanol at 4, 12 and 18 months of age. Trunk blood and brain tissue were collected immediately after euthanasia. Trunk blood was centrifuged for 15-20 min at 4°C x 14,500 RPM. Plasma was stored at -80C.

Blood Plasma and Brain homogenate analysis

Blood was collected from non-fasted mice aged 4, 12, 18 months of age. Mice were anesthetized and blood was extracted by left ventricle cardiac puncture with a 25 g EDTA-coated needle before PBS perfusion. Approximately 500 µL of whole blood was transferred to a MAP-K2 EDTA Microcontainer (BD, Franklin Lakes, NJ) on ice and centrifuged at 4°C x 4388 *g* in a pre-

bioRxiv preprint doi: https://doi.org/10.1101/2023.12.19.571985; this version posted December 20, 2023. The copyright holder for this preprint (which was not certified by peer review) is the author/funder, who has granted bioRxiv a license to display the preprint in perpetuity. It is made available under aCC-BY 4.0 International license.

chilled ultracentrifuge for 15 minutes. Without disturbing the red blood cell fraction, serum supernatant was pipetted into a chilled cryovial with a with p200 tip and immediately snap-frozen on dry ice for 10 min.

Brain homogenization and protein extraction: Hemibrains were homogenized in tissue homogenization buffer containing fresh protease inhibitor cocktail and aliquoted. DEA/Formic Acid extraction was carried out according to Casali et al., 2016. Supernatant was utilized for the cytokine analysis, soluble and insoluble components were used for the Aβ analysis.

Cytokine and Aβ Panel Assay: Mouse hemibrain samples were assayed in duplicate using the MSD mouse proinflammatory Panel I, a highly sensitive multiplex enzyme-linked immunosorbent assay (ELISA). The panel quantifies 10 cytokines: interferon γ (IFN-γ), interleukin (IL)-1β, IL-2, IL-4, IL-6, IL-8, IL-10, IL-12p70, IL-13, and tumor necrosis factor α (TNFα) from a single small sample volume (25 μL) using an electrochemiluminescent detection method (MesoScale Discovery, Gaithersburg, MD, USA). The mean intra-assay coefficient for each cytokine was <8.5%, based on cytokine standards. Any value that was below the lowest limit of detection (LLOD) for the cytokine assay was replaced with ½ LLOD of the assay for statistical analysis.

Aβ Species Assay: The plasma samples and soluble (DEA) components from hemibrain samples were assayed in duplicate using MSD Aβ Peptide Panel I (K15200E; MSD). Following the manufacturer's guidelines, the Aβ species (Aβ40 and Aβ42) were quantified from plasma and soluble component of hemibrain lysates using an electrochemiluminescent detection method (MSD). The levels of Aβ species from the assay were used for statistical analysis.

Immunohistochemistry

bioRxiv preprint doi: https://doi.org/10.1101/2023.12.19.571985; this version posted December 20, 2023. The copyright holder for this preprint (which was not certified by peer review) is the author/funder, who has granted bioRxiv a license to display the preprint in perpetuity. It is made available under aCC-BY 4.0 International license.

Left brain hemispheres were sectioned at 25 μm on a Thermo Scientific HM430 sliding microtome and blocked with 10% normal donkey or normal goat serum diluted in 1x PBS+0.5% TritonX wash buffer before immunohistochemical staining with antibodies selected to visualize neurons (NeuN), astrocytes (GFAP), microglia (Iba1), and nuclei (DAPI). Images were acquired at 20X using a Leica Aperio Versa (Germany) slide-scanner microscope. Cell counts, fluorescence intensity, and surface area ratios in the regions of the cortex and hippocampus were calculated using Imaris (Chicago, IL, USA).

Cohort 3 – Indiana University

To assess neurovascular coupling, mice were non-invasively imaged via PET/CT (n=12 mice/sex/genotype/age). Regional blood flow was be measured via ^{64}Cu-pyruvaldehyde-bis(N4-methylthiosemicarbazone) (^{64}Cu-PTSM) [56], which has a very high first pass (>75-90%) extraction[57], and glutathione reductase redox trapping of copper[57], was administered via tail vein in awake subjects, and allowed 2 min uptake period. To measure regional glycolytic metabolism, 2-^{18}F-2-deoxyglucose (^{18}F-FDG) was administered via inter-peritoneal injection in awake subjects and mice were given 30-45 min uptake period prior to imaging per our previous work [8]. Post uptake, mice were induced with 5% isoflurane (95% medical oxygen) and maintained with 1-2% isoflurane at 37°C. PET/CT imaging were performed with a Molecubes □-X-CUBE system (Molecubes NV), where calibrated listmode PET images were reconstructed into a single-static image previously described[8]. Helical CT images were also acquired for anatomical reference, and attenuation maps needed to correct PET images per our previous work[8]. PET and CT images were co-registered, and mapped to stereotactic mouse brain coordinates of Paxinos-Franklin[58]. To permit dose and scanner and brain uptake normalization, Standardized Uptake Value Ratios

bioRxiv preprint doi: https://doi.org/10.1101/2023.12.19.571985; this version posted December 20, 2023. The copyright holder for this preprint (which was not certified by peer review) is the author/funder, who has granted bioRxiv a license to display the preprint in perpetuity. It is made available under aCC-BY 4.0 International license.

(SUVR) relative to the cerebellum were computed for PET for each subject, genotype, and age as follows:

$$SUVR(s, R, g, a) = \frac{R(s, g, a)}{C(s, g, a)} \quad (1)$$

where, s, g, a, R, and C are the subject, genotype, age, region/volume of interest, cerebellum region/volume of interest. The SUVR values were then converted to z-score as follows:

$$z\ score(s, R, g, a) = \frac{SUVR(s, g, a) - \bar{X}_R(R, g, a)}{\sigma_R(R, g, a)} \quad (2)$$

where, s, g, a, R, $\bar{X}_R$, and σ_R are the subject, genotype, age, mean of the reference population in SUVR, standard deviation of the reference population, based on the specified analytical strategies (effects of aging, humanized genes, and AD-risk alleles). Data are then plotted on a Cartesian coordinate system, where [0,0] is no change in tissue perfusion (x-axis) or metabolism (y-axis) from the reference population.

Cryotomy

After PET imaging, animals were euthanized and brains were removed, bisected, and snap-frozen on dry ice before embedding in OCT in cryo-molds. Brains were sectioned at 20 µm with a Leica 1850 or CM1860 Cryotome, with 6 per slide per bregma region of interest. Bregmas: 0.38, -1.94, -3.8, -5.88. ROIs: Corpus Callosum, Striatum, Cing Ctx, Motor Ctx, Somatosensory Ctx, DI Ctx, Med Sep, Hypothalamus, Hippocampus, Retrosplenial Ctx, Auditory Ctx, Entrohinal Ctx, Thalamus, TEA, Visual Ctx, Cerebellum.

Autoradiography

bioRxiv preprint doi: https://doi.org/10.1101/2023.12.19.571985; this version posted December 20, 2023. The copyright holder for this preprint (which was not certified by peer review) is the author/funder, who has granted bioRxiv a license to display the preprint in perpetuity. It is made available under aCC-BY 4.0 International license.

Standards for calibration were prepared using two-fold serial dilutions of PET tracer in 3.2% low melt agarose, 4% sucrose, 0.2% gelatin. Aliquots (~30 µL) were dotted into cryomolds and allowed to firm up. OCT was added and standards were frozen and cut at the same thickness as the brain sections. An additional ~20 µL aliquot was pipetted into a 7 mL scintillation tube filled with scintillation fluid and counted on a Beckman LSC 6500 for 15 to 30 seconds using the wide window. These data were used to calculate the standard curve for each animal and were converted to Bq/ml using a standard calculation worksheet. Slides with brain sections were placed on cardboard along with standards and exposed overnight to phosphorimager before imaging on the GE Typhoon FLA7000IP.

Cohort 4 – University of Pittsburgh

Breeding and Husbandry

After weaning, all mice were housed by sex, 2-5 mice per cage with P.J. Murphy Coarse Certified Aspen Sani-Chip® bedding and initially provided LabDiet® 5P76 (6% fat; control diet, CD) *ad libitum* until 2 months of age. At 2 months of age, mice assigned to the HFD group were given *ad libitum* access to a 45% kcal fat rodent diet (Research Diets, D12451i). All cages included enrichment consisting of nestlets (Ancare, NES3600) and red PET plastic domes (Braintree Scientific). The mice were kept on a 12-hour light/dark schedule with the lights on from 7:00 a.m. to 7:00 p.m. daily. The room was maintained at 72-74°F and 30-70% humidity. Chlorinated water (2-4ppm; pH 4-5) was provided *ad libitum* via a lixit system. The animals were ear-punched for identification purposes and subsequently microchipped at the tail base using a p-chip system (PharmaSeq).

bioRxiv preprint doi: https://doi.org/10.1101/2023.12.19.571985; this version posted December 20, 2023. The copyright holder for this preprint (which was not certified by peer review) is the author/funder, who has granted bioRxiv a license to display the preprint in perpetuity. It is made available under aCC-BY 4.0 International license.

Plasma biomarkers

Cytokines

Subjects were evaluated monthly for plasma cytokines from 2-13 months of age and then at 15 and 18 months of age (Figure 8A). Plasma was also collected and analyzed at 8.5 and 10.5 months, each after 2-week bouts of food restriction as described in the following sections. For longitudinal analysis of plasma cytokines, 50µL blood was collected via the tail tip in non-anesthetized mice into heparinized capillary tubes and then transferred to chilled 1.5mL microtubes and centrifuged @4C for 10 min x 14500 rpm. Plasma was stored as 25µL aliquots at -80C until analysis. MesoScale Discovery multiplex ELISA kits were run according to the manufacturer's protocol for the mouse pro-inflammatory cytokine panel (Kit# K15048D).

Aβ40 and Aβ42

Subjects were evaluated at 2, 6, 9, 12, 15, and 18 months for plasma Aβ40 and 42 (Figure 8A). After a heparinized blood sample was collected for analysis of plasma cytokines, 40-50µL blood was collected via the same tail tip into EDTA coated capillary tubes and then transferred to chilled 1.5mL microtubes and centrifuged @4C for 10 min x 14500 rpm. Plasma was stored as 20µL aliquots at -80C until analysis. MesoScale Discovery (MSD) multiplex ELISA kits were run according to the manufacturer's protocol for the 6E10 Aβ peptide panel (Kit# K15200E).

Statistical Analysis of Plasma Biomarkers

MSD ELISA results were analyzed in Discovery Workbench Software provided by MSD. All samples from a single plasma collection timepoint were run together on the appropriate MSD

bioRxiv preprint doi: https://doi.org/10.1101/2023.12.19.571985; this version posted December 20, 2023. The copyright holder for this preprint (which was not certified by peer review) is the author/funder, who has granted bioRxiv a license to display the preprint in perpetuity. It is made available under aCC-BY 4.0 International license.

plate with calibration standards. The calibration standards from each plate and the accuracy and precision between and within runs were evaluated based on the FDA's M10 Bioanalytical Method Validation guidelines for ligand binding assays. Calibration standards whose back-calculated concentrations exceeded ±20% (or ±25% at the ULOQ and LLOQ) of the expected concentration (% recovery) were excluded. At least 75% of all calibration standards and at least 6 concentrations per assay were required to meet criteria for each plate to be included in statistical analysis. Across all plates run for each assay, the % recovery for each included calibration standard ranged from 95-105%.

After completion of within and between plate quality control, plasma biomarker concentrations for 8 cytokines (TNF-α, IL-6, IL-10, IL-1β, IFN-γ, IL-5, KC/GRO, and IL-2), Aβ40, and Aβ42 were compared using two-way ANOVA with Tukey's multiple comparisons test and a 95% confidence interval.

Food Restriction

Beginning at 8 months of age, mice were individually housed and restricted to 80-85% of free-feeding body weight (Figure 8A). Mice were weighed daily and provided a ration of the respective CD or HFD diets that maintained them at 80-85% restriction.

Touchscreen cognitive testing

Male and female LOAD2 mice exposed to ad libitum high fat diet (HFD) from 2 months of age (Figure 8A) and LOAD2 or C57BL/6J (WT) mice exposed to HFD from 6+ months of age (Supplemental Figure 7A) and age- and sex-matched LOAD2 and C57BL/6J WT controls

bioRxiv preprint doi: https://doi.org/10.1101/2023.12.19.571985; this version posted December 20, 2023. The copyright holder for this preprint (which was not certified by peer review) is the author/funder, who has granted bioRxiv a license to display the preprint in perpetuity. It is made available under aCC-BY 4.0 International license.

maintained on normal control diet (CD) chow were enrolled for cognitive testing beginning from after 12 months of age. Prior to enrollment blood was collected (non-anesthetized) up to twice per month via the tail collection method as described above; otherwise, subjects were behaviorally naïve prior to touchscreen task acquisition. Mice were trained and tested in daily sessions (typically 5-6 days per week) using the Bussey-Saksida mouse touchscreen chambers and ABET II software pre-programmed protocols for the Location Discrimination task (Lafayette Instrument Company, Lafayette, IN, USA) similar to the methods previously described for the location discrimination task[11]. Trained technicians were blinded to genotype throughout testing though blinding for diet was not possible due to visual differences in HFD v CD. Genotype, sex, and diet treatment were randomized and counterbalanced across multiple chambers and sessions though the same subject was always assigned to the same chamber throughout training and testing. The touchscreen chambers were enclosed in sound-attenuated and ventilated chambers and a black Perspex mask consisting of two rows, each of 6 adjacent square response windows placed directly in front of the touchscreen was used to isolate only the areas on the touchscreen that are relevant to the task stimuli presented and minimize non-targeted touch responses to the screen. During the pre-training phase, subjects were food restricted to 80-85% of free feeding body weight and acclimated to the reinforcer used for the training and testing in their homecages (10% sucrose solution formulated in drinking water). Once subjects were within the requisite restricted weight range, habituation and training using a step wise criterion-based approach was initiated similar to as previously described[11]). During the initial acquisition phase of the task, subjects were trained to associate nosepoke touches to an illuminated square on the screen with the presentation of a reward (20µL of 10% sucrose solution). Touch responses to blank squares had no programmed consequences. This ‘must

bioRxiv preprint doi: https://doi.org/10.1101/2023.12.19.571985; this version posted December 20, 2023. The copyright holder for this preprint (which was not certified by peer review) is the author/funder, who has granted bioRxiv a license to display the preprint in perpetuity. It is made available under aCC-BY 4.0 International license.

touch' phase was subsequently followed by a 'punish incorrect' phase in which touch responses to illuminated stimuli were rewarded, while touches to blank squares resulted in the illumination of the houselight with no reward delivered. Accuracy as an indicator of learning was analyzed during the punish incorrect phase of the training by two-way repeated measures ANOVA (treatment group x time). Subjects that failed to meet criterion during each step were not advanced for further cognitive testing. More specifically, subjects that failed to meet task acquisition criteria during the punish incorrect phase were not assessed for pattern separation in the location discrimination (LD) task. For subjects that advanced to the LD task, during LD acquisition trials only the bottom row of 6 adjacent squares was used and annotated for description purposes as squares 1-6 from left to right (see Oomen et al 2013). During the LD training phase, only squares 2 and 5 were used that represent an intermediate location separation for left and right positions, respectively on the screen. During the Intermediate sessions, both left and right squares were illuminated with one designated as correct and the other as incorrect. A nosepoke response made to the correct location is rewarded while incorrect responses result in a 5 second timeout period paired with the houselight turning on. After a 10 second inter-trial interval (ITI), both stimuli in the identical locations is presented again. Once the subject makes 7 correct out of 8 consecutive responses, then the rule is reversed and the subject must determine through trial and error the correct rewarded location. For each consecutive session, the location (left or right) assigned as correct at the start of the session was reversed to avoid mediating strategies. A priori criterion to advance to further pattern separation assessments is 2 consecutive days with ≥3 serial reversals within a session (max trials allowed, max session time limit = 60 min). Upon meeting criteria during the intermediate phase of the test, subjects are evaluated in 2 consecutive sessions with the locations of the two stimuli with the furthest separation = "easy"

bioRxiv preprint doi: https://doi.org/10.1101/2023.12.19.571985; this version posted December 20, 2023. The copyright holder for this preprint (which was not certified by peer review) is the author/funder, who has granted bioRxiv a license to display the preprint in perpetuity. It is made available under aCC-BY 4.0 International license.

difficulty (positions 1 and 6) followed by 2 consecutive sessions with the closest separation of the two stimuli = hard difficulty (positions 3 and 4). The primary measures of performance are the # trials to criterion.

Cohort 5 – University of Pittsburgh

Breeding and Husbandry

After weaning, all mice were housed by sex, 2-4 mice per cage with P.J. Murphy Coarse Certified Aspen Sani-Chip® bedding and initially provided LabDiet® 5P76 (6% fat; control diet, CD) *ad libitum* until 6-12 months of age. At 6-12 months of age, mice assigned to the HFD group were given *ad libitum* access to a 45% kcal fat rodent diet (Research Diets, D12451i). All cages included enrichment in the form of nestlets (Ancare, NES3600) and red PET plastic domes (Braintree Scientific). The mice were kept on a 12-hour light/dark schedule with the lights on from 7:00 a.m. to 7:00 p.m. daily. The room was maintained at 72-74°F and 40-60% humidity. Chlorinated water (2-3ppm; pH 4-5) was provided *ad libitum* via a lixit system. The animals were ear-punched for identification purposes and subsequently microchipped at the tail base using a p-chip system (PharmaSeq). Prior to beginning touchscreen training at 11-17 months of age (Supplementary Fig 7A), mice were individually housed and restricted to 80-85% of free-feeding body weight. Mice were weighed daily and provided a ration of the respective CD or HFD diets that maintained them at 80-85% restriction.

Plasma biomarkers

bioRxiv preprint doi: https://doi.org/10.1101/2023.12.19.571985; this version posted December 20, 2023. The copyright holder for this preprint (which was not certified by peer review) is the author/funder, who has granted bioRxiv a license to display the preprint in perpetuity. It is made available under aCC-BY 4.0 International license.

Cytokines

Subjects were evaluated monthly for plasma cytokines from 6-12 until 11-17 months of age (Supplemental Figure 7A). Collection, storage, and analysis of plasma for proinflammatory cytokines followed the methods described above for Cohort 4.

Aβ40 and Aβ42

Subjects were evaluated at a single timepoint, 11-17 months, for plasma Aβ40 and 42 (Supplemental Figure 7A). Collection, storage, and analysis of plasma for Aβ species followed the methods described above for Cohort 4.

bioRxiv preprint doi: https://doi.org/10.1101/2023.12.19.571985; this version posted December 20, 2023. The copyright holder for this preprint (which was not certified by peer review) is the author/funder, who has granted bioRxiv a license to display the preprint in perpetuity. It is made available under aCC-BY 4.0 International license.

References

1. Baumgart M, Snyder HM, Carrillo MC, Fazio S, Kim H, Johns H. Summary of the evidence on modifiable risk factors for cognitive decline and dementia: A population-based perspective. Alzheimers Dement. 2015;11(6):718-26. Epub 20150601. doi: 10.1016/j.jalz.2015.05.016. PubMed PMID: 26045020.
2. Boyd RJ, Avramopoulos D, Jantzie LL, McCallion AS. Neuroinflammation represents a common theme amongst genetic and environmental risk factors for Alzheimer and Parkinson diseases. J Neuroinflammation. 2022;19(1):223. Epub 20220908. doi: 10.1186/s12974-022-02584-x. PubMed PMID: 36076238; PMCID: PMC9452283.
3. Solfrizzi V, Panza F, Frisardi V, Seripa D, Logroscino G, Imbimbo BP, Pilotto A. Diet and Alzheimer's disease risk factors or prevention: the current evidence. Expert Rev Neurother. 2011;11(5):677-708. doi: 10.1586/ern.11.56. PubMed PMID: 21539488.
4. Biogen. FDA Approves Updated ADUHELM™ Prescribing Information to Emphasize Population Studied in Clinical Trials 2021 [cited 2023 11/21]. Available from: https://investors.biogen.com/news-releases/news-release-details/fda-approves-updated-aduhelmtm-prescribing-information-emphasize.
5. FDA. FDA Converts Novel Alzheimer's Disease Treatment to Traditional Approval 2023 [cited 2023 11/21]. Available from: https://www.fda.gov/news-events/press-announcements/fda-converts-novel-alzheimers-disease-treatment-traditional-approval.
6. Oblak AL, Forner S, Territo PR, Sasner M, Carter GW, Howell GR, Sukoff-Rizzo SJ, Logsdon BA, Mangravite LM, Mortazavi A, Baglietto-Vargas D, Green KN, MacGregor GR, Wood MA, Tenner AJ, LaFerla FM, Lamb BT, and The M-A, Consortium. Model organism development and evaluation for late-onset Alzheimer's disease: MODEL-AD. Alzheimers Dement (N Y). 2020;6(1):e12110. Epub 2020/12/08. doi: 10.1002/trc2.12110. PubMed PMID: 33283040; PMCID: 7683958.
7. Foley KE, Hewes AA, Garceau DT, Kotredes KP, Carter GW, Sasner M, Howell GR. The APOE (epsilon3/epsilon4) Genotype Drives Distinct Gene Signatures in the Cortex of Young Mice. Front Aging Neurosci. 2022;14:838436. Epub 20220316. doi: 10.3389/fnagi.2022.838436. PubMed PMID: 35370604; PMCID: PMC8967347.
8. Kotredes KP, Oblak AL, Pandey RS, Lin PB, Garceau D, Williams HM, Uyar A, O'Rourke R, O'Rourke S, Ingraham C, Bednarczyk D, Belanger M, Cope ZA, Foley KE, Logsdon BA, Mangravite LM, Sukoff Rizzo SJ, Territo PR, Carter GW, Sasner M, Lamb BT, Howell GR. Uncovering disease mechanisms in a novel mouse model expressing humanized APOEε4 and Trem2*R47H. Frontiers in Aging Neuroscience. 2021.
9. Morris MC, Evans DA, Bienias JL, Tangney CC, Bennett DA, Aggarwal N, Schneider J, Wilson RS. Dietary fats and the risk of incident Alzheimer disease. Arch Neurol. 2003;60(2):194-200. doi: 10.1001/archneur.60.2.194. PubMed PMID: 12580703.
10. Oblak AL, Kotredes KP, Pandey RS, Reagan AM, Ingraham C, Perkins B, Lloyd C, Baker D, Lin PB, Soni DM, Tsai AP, Persohn SA, Bedwell AA, Eldridge K, Speedy R, Meyer JA, Peters JS, Figueiredo LL, Sasner M, Territo PR, Sukoff Rizzo SJ, Carter GW, Lamb BT, Howell GR. Plcg2(M28L) Interacts With High Fat/High Sugar Diet to Accelerate Alzheimer's Disease-Relevant Phenotypes in Mice. Front Aging Neurosci. 2022;14:886575. Epub 20220624. doi: 10.3389/fnagi.2022.886575. PubMed PMID: 35813947; PMCID: PMC9263289.
11. Oomen CA, Hvoslef-Eide M, Heath CJ, Mar AC, Horner AE, Bussey TJ, Saksida LM. The touchscreen operant platform for testing working memory and pattern separation in rats and mice. Nat Protoc. 2013;8(10):2006-21. Epub 20130919. doi: 10.1038/nprot.2013.124. PubMed PMID: 24051961; PMCID: PMC3982138.

bioRxiv preprint doi: https://doi.org/10.1101/2023.12.19.571985; this version posted December 20, 2023. The copyright holder for this preprint (which was not certified by peer review) is the author/funder, who has granted bioRxiv a license to display the preprint in perpetuity. It is made available under aCC-BY 4.0 International license.

12. Ano Y, Nakayama H. Preventive Effects of Dairy Products on Dementia and the Underlying Mechanisms. Int J Mol Sci. 2018;19(7). Epub 20180630. doi: 10.3390/ijms19071927. PubMed PMID: 29966358; PMCID: PMC6073537.
13. Li Y, Zhang ZH, Huang SL, Yue ZB, Yin XS, Feng ZQ, Zhang XG, Song GL. Whey protein powder with milk fat globule membrane attenuates Alzheimer's disease pathology in 3xTg-AD mice by modulating neuroinflammation through the peroxisome proliferator-activated receptor gamma signaling pathway. J Dairy Sci. 2023;106(8):5253-65. Epub 20230704. doi: 10.3168/jds.2023-23254. PubMed PMID: 37414601.
14. Min LJ, Kobayashi Y, Mogi M, Tsukuda K, Yamada A, Yamauchi K, Abe F, Iwanami J, Xiao JZ, Horiuchi M. Administration of bovine casein-derived peptide prevents cognitive decline in Alzheimer disease model mice. PLoS One. 2017;12(2):e0171515. Epub 20170203. doi: 10.1371/journal.pone.0171515. PubMed PMID: 28158298; PMCID: PMC5291428.
15. Sukoff Rizzo SJ, Anderson LC, Green TL, McGarr T, Wells G, Winter SS. Assessing Healthspan and Lifespan Measures in Aging Mice: Optimization of Testing Protocols, Replicability, and Rater Reliability. Curr Protoc Mouse Biol. 2018;8(2):e45. doi: 10.1002/cpmo.45. PubMed PMID: 29924918.
16. Backstrom D, Linder J, Jakobson Mo S, Riklund K, Zetterberg H, Blennow K, Forsgren L, Lenfeldt N. NfL as a biomarker for neurodegeneration and survival in Parkinson disease. Neurology. 2020;95(7):e827-e38. Epub 20200717. doi: 10.1212/WNL.0000000000010084. PubMed PMID: 32680941; PMCID: PMC7605503.
17. Langfelder P, Horvath S. WGCNA: an R package for weighted correlation network analysis. BMC Bioinformatics. 2008;9:559. Epub 2008/12/31. doi: 10.1186/1471-2105-9-559. PubMed PMID: 19114008; PMCID: PMC2631488.
18. Cary GA, Wiley JC, Gockley J, Keegan S, Heath L, R. R. Butler I, Mangravite LM, Logsdon BA, Longo FM, Levey A, Greenwood AK, Carter GW, The Emory-Sage SGCT-ADC. Genetic and Multi-omic Risk Assessment of Alzheimer's Disease Implicates Core Associated Biological Domains. medRxiv. 2022:2022.12.15.22283478. doi: 10.1101/2022.12.15.22283478.
19. Johnson ECB, Carter EK, Dammer EB, Duong DM, Gerasimov ES, Liu Y, Liu J, Betarbet R, Ping L, Yin L, Serrano GE, Beach TG, Peng J, De Jager PL, Haroutunian V, Zhang B, Gaiteri C, Bennett DA, Gearing M, Wingo TS, Wingo AP, Lah JJ, Levey AI, Seyfried NT. Large-scale deep multi-layer analysis of Alzheimer's disease brain reveals strong proteomic disease-related changes not observed at the RNA level. Nat Neurosci. 2022;25(2):213-25. Epub 20220203. doi: 10.1038/s41593-021-00999-y. PubMed PMID: 35115731; PMCID: PMC8825285.
20. Dong H, Csernansky JG. Effects of stress and stress hormones on amyloid-beta protein and plaque deposition. J Alzheimers Dis. 2009;18(2):459-69. doi: 10.3233/jad-2009-1152. PubMed PMID: 19584430; PMCID: PMC2905685.
21. Milind N, Preuss C, Haber A, Ananda G, Mukherjee S, John C, Shapley S, Logsdon BA, Crane PK, Carter GW. Transcriptomic stratification of late-onset Alzheimer's cases reveals novel genetic modifiers of disease pathology. PLoS Genet. 2020;16(6):e1008775. Epub 20200603. doi: 10.1371/journal.pgen.1008775. PubMed PMID: 32492070; PMCID: PMC7295244.
22. Mukherjee S, Heath L, Preuss C, Jayadev S, Garden GA, Greenwood AK, Sieberts SK, De Jager PL, Ertekin-Taner N, Carter GW, Mangravite LM, Logsdon BA. Molecular estimation of neurodegeneration pseudotime in older brains. Nat Commun. 2020;11(1):5781. Epub 20201113. doi: 10.1038/s41467-020-19622-y. PubMed PMID: 33188183; PMCID: PMC7666177.
23. Naj AC, Schellenberg GD. Genomic variants, genes, and pathways of Alzheimer's disease: An overview. Am J Med Genet B Neuropsychiatr Genet. 2017;174(1):5-26. doi: 10.1002/ajmg.b.32499. PubMed PMID: 27943641; PMCID: PMC6179157.

bioRxiv preprint doi: https://doi.org/10.1101/2023.12.19.571985; this version posted December 20, 2023. The copyright holder for this preprint (which was not certified by peer review) is the author/funder, who has granted bioRxiv a license to display the preprint in perpetuity. It is made available under aCC-BY 4.0 International license.

24. Verheijen J, Sleegers K. Understanding Alzheimer Disease at the Interface between Genetics and Transcriptomics. Trends Genet. 2018;34(6):434-47. Epub 20180321. doi: 10.1016/j.tig.2018.02.007. PubMed PMID: 29573818.
25. Balusu S, Horre K, Thrupp N, Craessaerts K, Snellinx A, Serneels L, T'Syen D, Chrysidou I, Arranz AM, Sierksma A, Simren J, Karikari TK, Zetterberg H, Chen WT, Thal DR, Salta E, Fiers M, De Strooper B. MEG3 activates necroptosis in human neuron xenografts modeling Alzheimer's disease. Science. 2023;381(6663):1176-82. Epub 20230914. doi: 10.1126/science.abp9556. PubMed PMID: 37708272; PMCID: PMC7615236.
26. Urai AE, Aguillon-Rodriguez V, Laranjeira IC, Cazettes F, International Brain L, Mainen ZF, Churchland AK. Citric Acid Water as an Alternative to Water Restriction for High-Yield Mouse Behavior. eNeuro. 2021;8(1). Epub 20210211. doi: 10.1523/ENEURO.0230-20.2020. PubMed PMID: 33431508; PMCID: PMC7890523.
27. Bennett DA. Mixed pathologies and neural reserve: Implications of complexity for Alzheimer disease drug discovery. PLoS Med. 2017;14(3):e1002256. Epub 20170314. doi: 10.1371/journal.pmed.1002256. PubMed PMID: 28291788; PMCID: PMC5349649.
28. Girouard H, Iadecola C. Neurovascular coupling in the normal brain and in hypertension, stroke, and Alzheimer disease. J Appl Physiol (1985). 2006;100(1):328-35. doi: 10.1152/japplphysiol.00966.2005. PubMed PMID: 16357086.
29. Kalaria RN, Erkinjuntti T. Small vessel disease and subcortical vascular dementia. J Clin Neurol. 2006;2(1):1-11. Epub 20060320. doi: 10.3988/jcn.2006.2.1.1. PubMed PMID: 20396480; PMCID: PMC2854938.
30. Liu CC, Liu CC, Kanekiyo T, Xu H, Bu G. Apolipoprotein E and Alzheimer disease: risk, mechanisms and therapy. Nat Rev Neurol. 2013;9(2):106-18. Epub 20130108. doi: 10.1038/nrneurol.2012.263. PubMed PMID: 23296339; PMCID: PMC3726719.
31. Bell RD, Winkler EA, Singh I, Sagare AP, Deane R, Wu Z, Holtzman DM, Betsholtz C, Armulik A, Sallstrom J, Berk BC, Zlokovic BV. Apolipoprotein E controls cerebrovascular integrity via cyclophilin A. Nature. 2012;485(7399):512-6. Epub 20120516. doi: 10.1038/nature11087. PubMed PMID: 22622580; PMCID: PMC4047116.
32. Bomfim TR, Forny-Germano L, Sathler LB, Brito-Moreira J, Houzel JC, Decker H, Silverman MA, Kazi H, Melo HM, McClean PL, Holscher C, Arnold SE, Talbot K, Klein WL, Munoz DP, Ferreira ST, De Felice FG. An anti-diabetes agent protects the mouse brain from defective insulin signaling caused by Alzheimer's disease- associated Abeta oligomers. J Clin Invest. 2012;122(4):1339-53. doi: 10.1172/JCI57256. PubMed PMID: 22476196; PMCID: PMC3314445.
33. Ferreira ST, Clarke JR, Bomfim TR, De Felice FG. Inflammation, defective insulin signaling, and neuronal dysfunction in Alzheimer's disease. Alzheimers Dement. 2014;10(1 Suppl):S76-83. doi: 10.1016/j.jalz.2013.12.010. PubMed PMID: 24529528.
34. Yu J, deMuinck ED, Zhuang Z, Drinane M, Kauser K, Rubanyi GM, Qian HS, Murata T, Escalante B, Sessa WC. Endothelial nitric oxide synthase is critical for ischemic remodeling, mural cell recruitment, and blood flow reserve. Proc Natl Acad Sci U S A. 2005;102(31):10999-1004. Epub 20050725. doi: 10.1073/pnas.0501444102. PubMed PMID: 16043715; PMCID: PMC1182413.
35. Salvadó G, Milà-Alomà M, Shekari M, Ashton NJ, Operto G, Falcon C, Cacciaglia R, Minguillon C, Fauria K, Niñerola-Baizán A, Perissinotti A, Benedet AL, Kollmorgen G, Suridjan I, Wild N, Molinuevo JL, Zetterberg H, Blennow K, Suárez-Calvet M, Gispert JD. Reactive astrogliosis is associated with higher cerebral glucose consumption in the early Alzheimer's continuum. Eur J Nucl Med Mol Imaging. 2022;49(13):4567-79. Epub 20220718. doi: 10.1007/s00259-022-05897-4. PubMed PMID: 35849149; PMCID: PMC9606048.

bioRxiv preprint doi: https://doi.org/10.1101/2023.12.19.571985; this version posted December 20, 2023. The copyright holder for this preprint (which was not certified by peer review) is the author/funder, who has granted bioRxiv a license to display the preprint in perpetuity. It is made available under aCC-BY 4.0 International license.

36. Ashraf A, Fan Z, Brooks DJ, Edison P. Cortical hypermetabolism in MCI subjects: a compensatory mechanism? Eur J Nucl Med Mol Imaging. 2015;42(3):447-58. Epub 20140930. doi: 10.1007/s00259-014-2919-z. PubMed PMID: 25267349.
37. Rubinski A, Franzmeier N, Neitzel J, Ewers M. FDG-PET hypermetabolism is associated with higher tau-PET in mild cognitive impairment at low amyloid-PET levels. Alzheimers Res Ther. 2020;12(1):133. Epub 20201019. doi: 10.1186/s13195-020-00702-6. PubMed PMID: 33076977; PMCID: PMC7574434.
38. Willette AA, Modanlo N, Kapogiannis D. Insulin resistance predicts medial temporal hypermetabolism in mild cognitive impairment conversion to Alzheimer disease. Diabetes. 2015;64(6):1933-40. Epub 20150109. doi: 10.2337/db14-1507. PubMed PMID: 25576061; PMCID: PMC4439566.
39. Yu L, Jin J, Xu Y, Zhu X. Aberrant Energy Metabolism in Alzheimer's Disease. J Transl Int Med. 2022;10(3):197-206. Epub 20220924. doi: 10.2478/jtim-2022-0024. PubMed PMID: 36776238; PMCID: PMC9901551.
40. Thomas KR, Osuna JR, Weigand AJ, Edmonds EC, Clark AL, Holmqvist S, Cota IH, Wierenga CE, Bondi MW, Bangen KJ. Regional hyperperfusion in older adults with objectively-defined subtle cognitive decline. J Cereb Blood Flow Metab. 2021;41(5):1001-12. Epub 20200702. doi: 10.1177/0271678x20935171. PubMed PMID: 32615887; PMCID: PMC8054731.
41. Dobin A, Davis CA, Schlesinger F, Drenkow J, Zaleski C, Jha S, Batut P, Chaisson M, Gingeras TR. STAR: ultrafast universal RNA-seq aligner. Bioinformatics. 2012;29(1):15-21. doi: 10.1093/bioinformatics/bts635.
42. Li B, Dewey CN. RSEM: accurate transcript quantification from RNA-Seq data with or without a reference genome. BMC Bioinformatics. 2011;12(1):323. doi: 10.1186/1471-2105-12-323.
43. Love MI, Huber W, Anders S. Moderated estimation of fold change and dispersion for RNA-seq data with DESeq2. Genome Biology. 2014;15(12):550. doi: 10.1186/s13059-014-0550-8.
44. Yu G, Wang L-G, Han Y, He Q-Y. clusterProfiler: an R Package for Comparing Biological Themes Among Gene Clusters. OMICS : a Journal of Integrative Biology. 2012;16(5):284-7. doi: 10.1089/omi.2011.0118. PubMed PMID: PMC3339379.
45. Zhang B, Horvath S. A general framework for weighted gene co-expression network analysis. Stat Appl Genet Mol Biol. 2005;4:Article17. Epub 2006/05/02. doi: 10.2202/1544-6115.1128. PubMed PMID: 16646834.
46. Ping L, Kundinger SR, Duong DM, Yin L, Gearing M, Lah JJ, Levey AI, Seyfried NT. Global quantitative analysis of the human brain proteome and phosphoproteome in Alzheimer's disease. Sci Data. 2020;7(1):315. Epub 2020/09/29. doi: 10.1038/s41597-020-00650-8. PubMed PMID: 32985496; PMCID: PMC7522715.
47. Kong AT, Leprevost FV, Avtonomov DM, Mellacheruvu D, Nesvizhskii AI. MSFragger: ultrafast and comprehensive peptide identification in mass spectrometry-based proteomics. Nat Methods. 2017;14(5):513-20. Epub 2017/04/11. doi: 10.1038/nmeth.4256. PubMed PMID: 28394336; PMCID: PMC5409104.
48. Yu F, Teo GC, Kong AT, Haynes SE, Avtonomov DM, Geiszler DJ, Nesvizhskii AI. Identification of modified peptides using localization-aware open search. Nat Commun. 2020;11(1):4065. Epub 2020/08/15. doi: 10.1038/s41467-020-17921-y. PubMed PMID: 32792501; PMCID: PMC7426425.
49. da Veiga Leprevost F, Haynes SE, Avtonomov DM, Chang H-Y, Shanmugam AK, Mellacheruvu D, Kong AT, Nesvizhskii AI. Philosopher: a versatile toolkit for shotgun proteomics data analysis. Nature Methods. 2020;17(9):869-70. doi: 10.1038/s41592-020-0912-y.
50. Kall L, Canterbury JD, Weston J, Noble WS, MacCoss MJ. Semi-supervised learning for peptide identification from shotgun proteomics datasets. Nat Methods. 2007;4(11):923-5. Epub 2007/10/24. doi: 10.1038/nmeth1113. PubMed PMID: 17952086.

bioRxiv preprint doi: https://doi.org/10.1101/2023.12.19.571985; this version posted December 20, 2023. The copyright holder for this preprint (which was not certified by peer review) is the author/funder, who has granted bioRxiv a license to display the preprint in perpetuity. It is made available under aCC-BY 4.0 International license.

51. Dammer EB, Seyfried NT, Johnson ECB. Batch Correction and Harmonization of -Omics Datasets with a Tunable Median Polish of Ratio. Front Syst Biol. 2023;3. Epub 2023/05/01. doi: 10.3389/fsysb.2023.1092341. PubMed PMID: 37122388; PMCID: PMC10137904.
52. Pandey RS, Graham L, Uyar A, Preuss C, Howell GR, Carter GW. Genetic perturbations of disease risk genes in mice capture transcriptomic signatures of late-onset Alzheimer's disease. Mol Neurodegener. 2019;14(1):50. Epub 2019/12/28. doi: 10.1186/s13024-019-0351-3. PubMed PMID: 31878951; PMCID: PMC6933917.
53. Preuss C, Pandey R, Piazza E, Fine A, Uyar A, Perumal T, Garceau D, Kotredes KP, Williams H, Mangravite LM, Lamb BT, Oblak AL, Howell GR, Sasner M, Logsdon BA, Consortium M-A, Carter GW. A novel systems biology approach to evaluate mouse models of late-onset Alzheimer's disease. Mol Neurodegener. 2020;15(1):67. Epub 2020/11/12. doi: 10.1186/s13024-020-00412-5. PubMed PMID: 33172468; PMCID: PMC7656729.
54. Ronneberger O, Fischer P, Brox T. U-Net: Convolutional Networks for Biomedical Image Segmentation. Springer International Publishing; 2015. p. 234-41.
55. Avants BB, Epstein CL, Grossman M, Gee JC. Symmetric diffeomorphic image registration with cross-correlation: evaluating automated labeling of elderly and neurodegenerative brain. Med Image Anal. 2008;12(1):26-41. Epub 20070623. doi: 10.1016/j.media.2007.06.004. PubMed PMID: 17659998; PMCID: PMC2276735.
56. Green MA. A potential copper radiopharmaceutical for imaging the heart and brain: copper-labeled pyruvaldehyde bis(N4-methylthiosemicarbazone). Int J Rad Appl Instrum B. 1987;14(1):59-61. Epub 1987/01/01. PubMed PMID: 3583756.
57. Mathias CJ, Welch MJ, Perry DJ, Mcguire AH, Zhu X, Connett JM, Green MA. Investigation of Copper-Ptsm as a Pet Tracer for Tumor Blood-Flow. Nuclear Medicine and Biology. 1991;18(7):807-11. PubMed PMID: WOS:A1991GF95400021.
58. Paxinos G, Franklin K. Paxinos and Franklin's the Mouse Brain in Stereotaxic Coordinates. New York, NY: Academic Press; 2012. 360 p.

bioRxiv preprint doi: https://doi.org/10.1101/2023.12.19.571985; this version posted December 20, 2023. The copyright holder for this preprint (which was not certified by peer review) is the author/funder, who has granted bioRxiv a license to display the preprint in perpetuity. It is made available under aCC-BY 4.0 International license.

bioRxiv preprint doi: https://doi.org/10.1101/2023.12.19.571985; this version posted December 20, 2023. The copyright holder for this preprint (which was not certified by peer review) is the author/funder, who has granted bioRxiv a license to display the preprint in perpetuity. It is made available under aCC-BY 4.0 International license.

bioRxiv preprint doi: https://doi.org/10.1101/2023.12.19.571985; this version posted December 20, 2023. The copyright holder for this preprint (which was not certified by peer review) is the author/funder, who has granted bioRxiv a license to display the preprint in perpetuity. It is made available under a CC-BY 4.0 International license.

SUPPLEMENTAL FIGURE 1

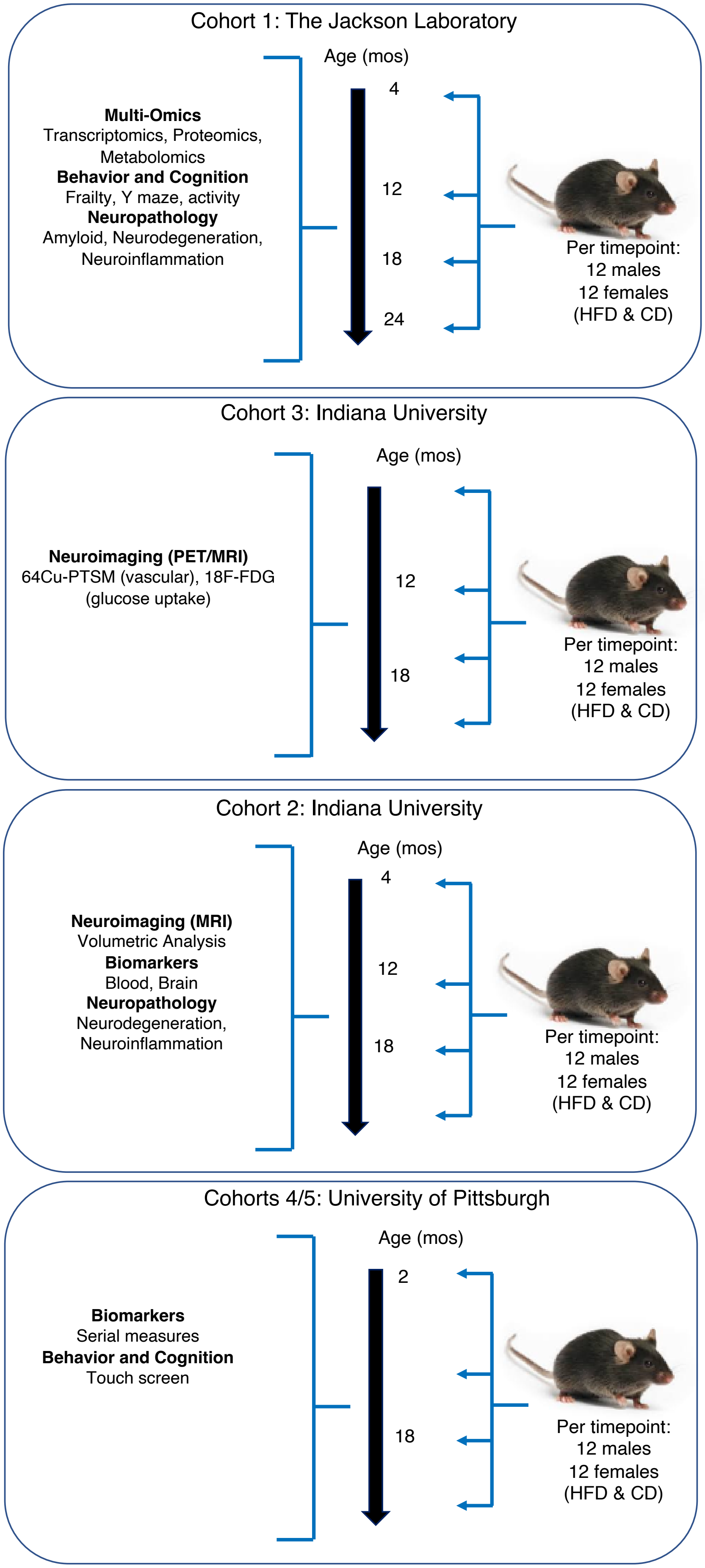

bioRxiv preprint doi: https://doi.org/10.1101/2023.12.19.571985; this version posted December 20, 2023. The copyright holder for this preprint (which was not certified by peer review) is the author/funder, who has granted bioRxiv a license to display the preprint in perpetuity. It is made available under aCC-BY 4.0 International license.

SUPPLEMENTAL FIGURE 2

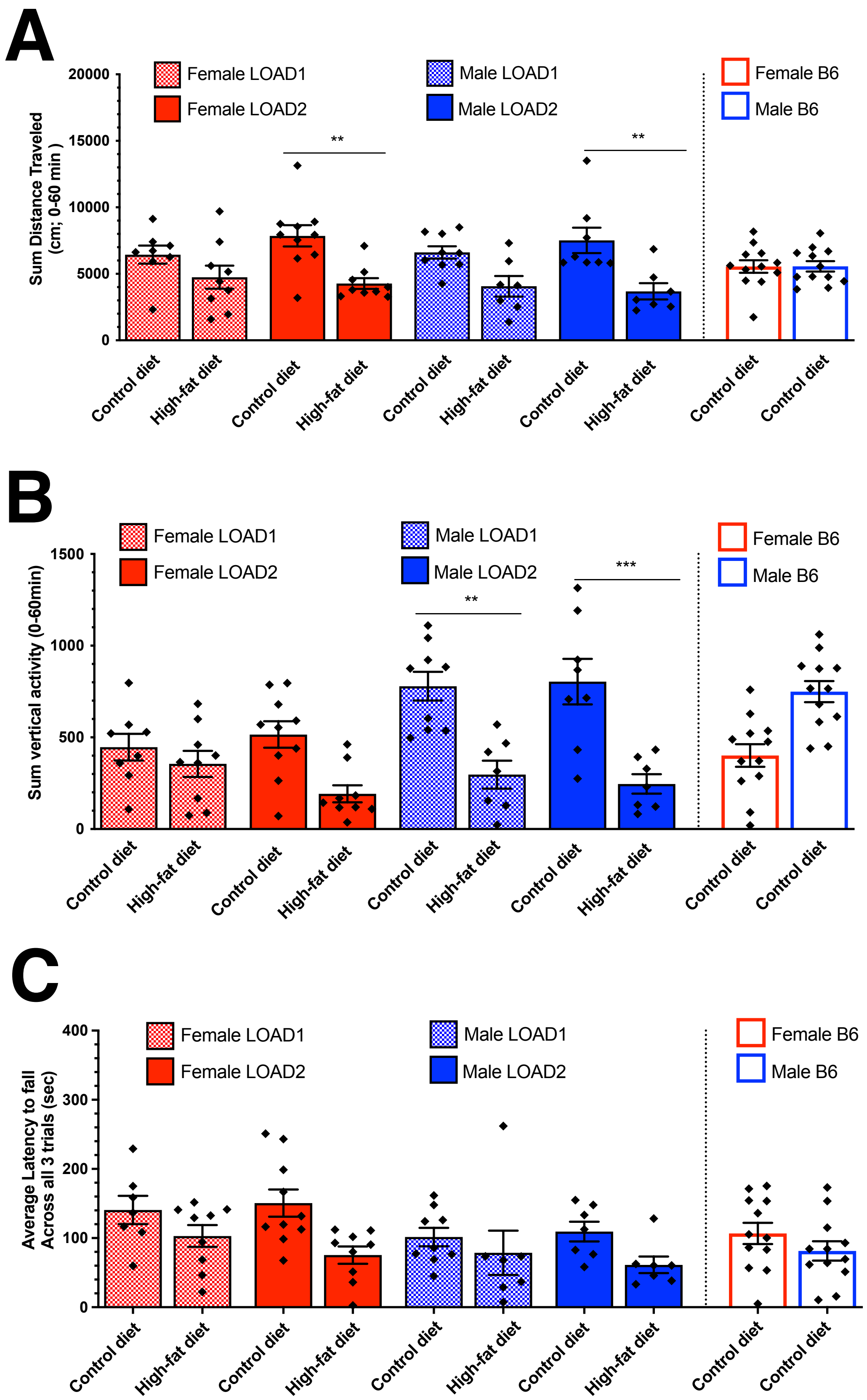

bioRxiv preprint doi: https://doi.org/10.1101/2023.12.19.571985; this version posted December 20, 2023. The copyright holder for this preprint (which was not certified by peer review) is the author/funder, who has granted bioRxiv a license to display the preprint in perpetuity. It is made available under aCC-BY 4.0 International license.

SUPPLEMENTAL FIGURE 3

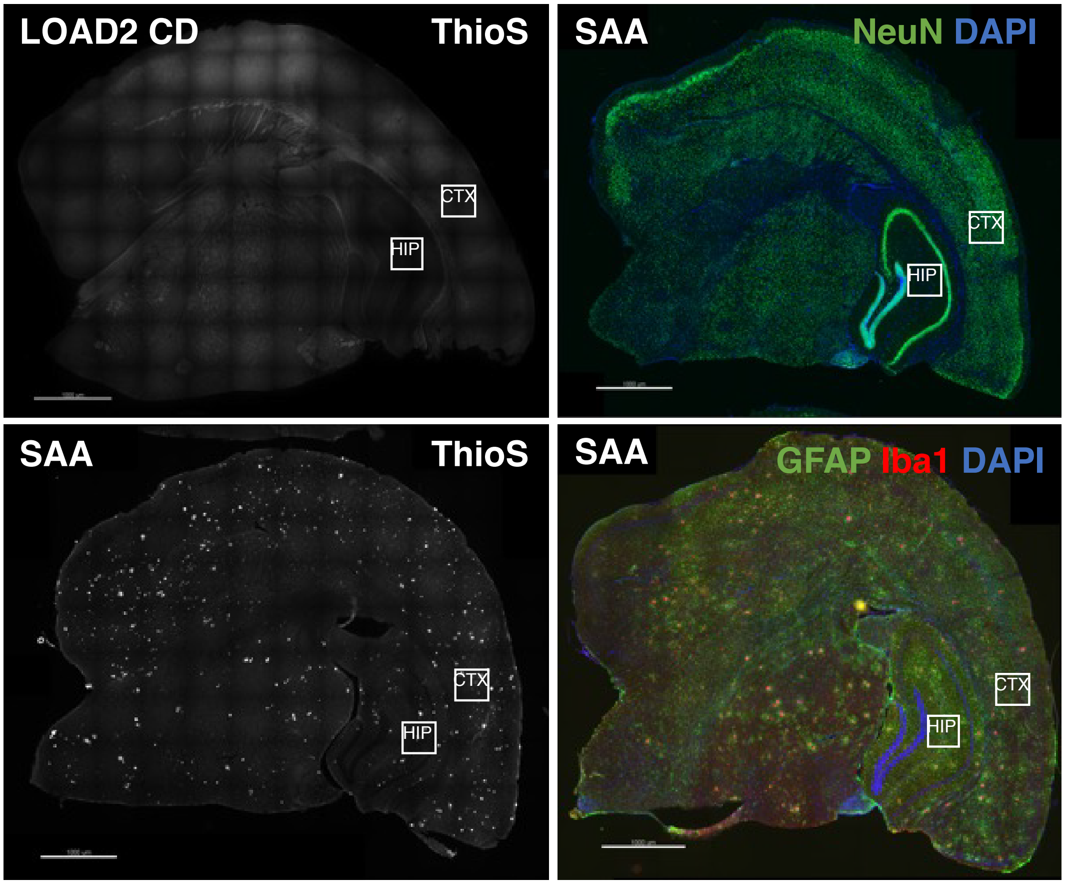

bioRxiv preprint doi: https://doi.org/10.1101/2023.12.19.571985; this version posted December 20, 2023. The copyright holder for this preprint (which was not certified by peer review) is the author/funder, who has granted bioRxiv a license to display the preprint in perpetuity. It is made available under aCC-BY 4.0 International license.

SUPPLEMENTAL FIGURE 4

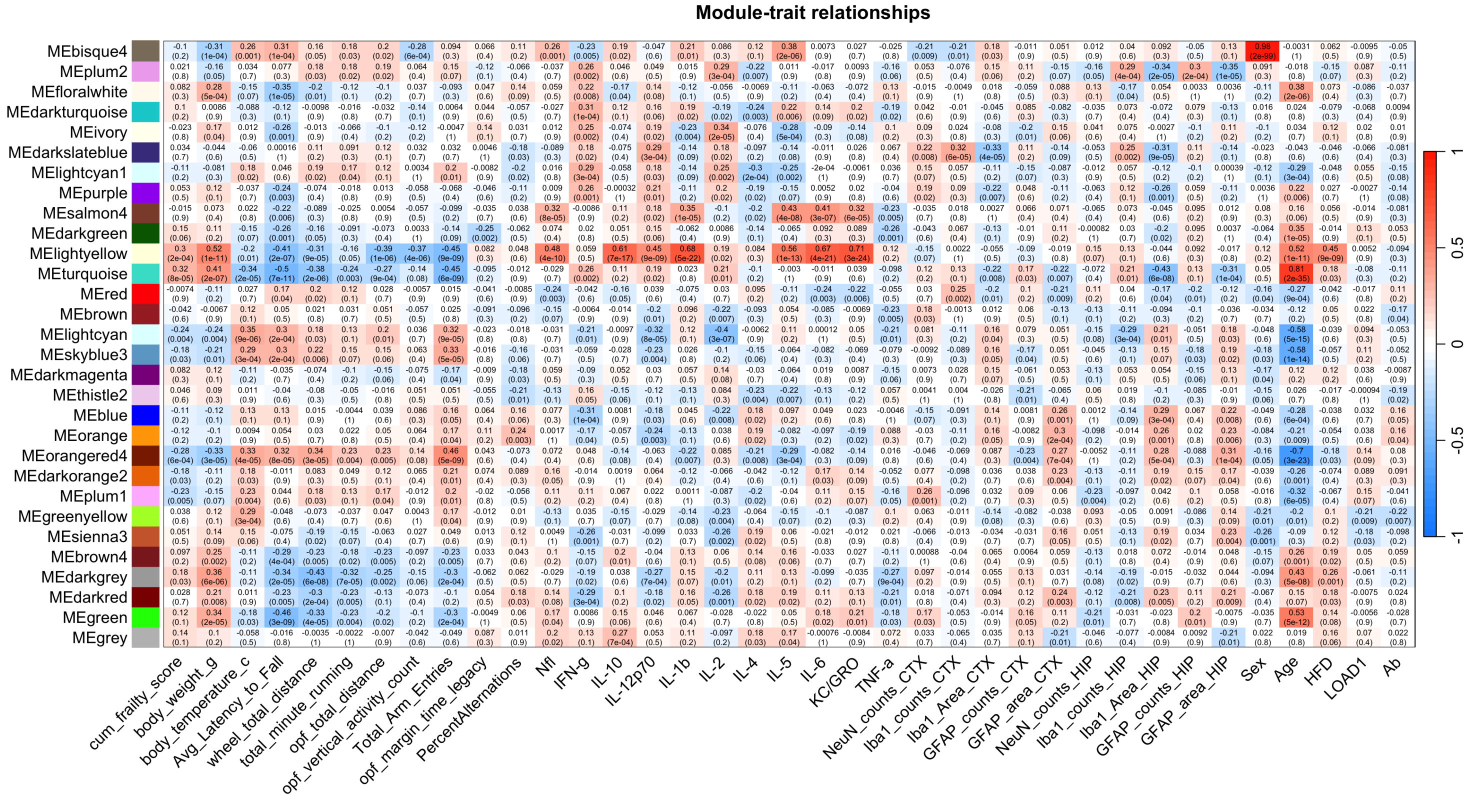

bioRxiv preprint doi: https://doi.org/10.1101/2023.12.19.571985; this version posted December 20, 2023. The copyright holder for this preprint (which was not certified by peer review) is the author/funder, who has granted bioRxiv a license to display the preprint in perpetuity. It is made available under aCC-BY 4.0 International license.

SUPPLEMENTAL FIGURE 5

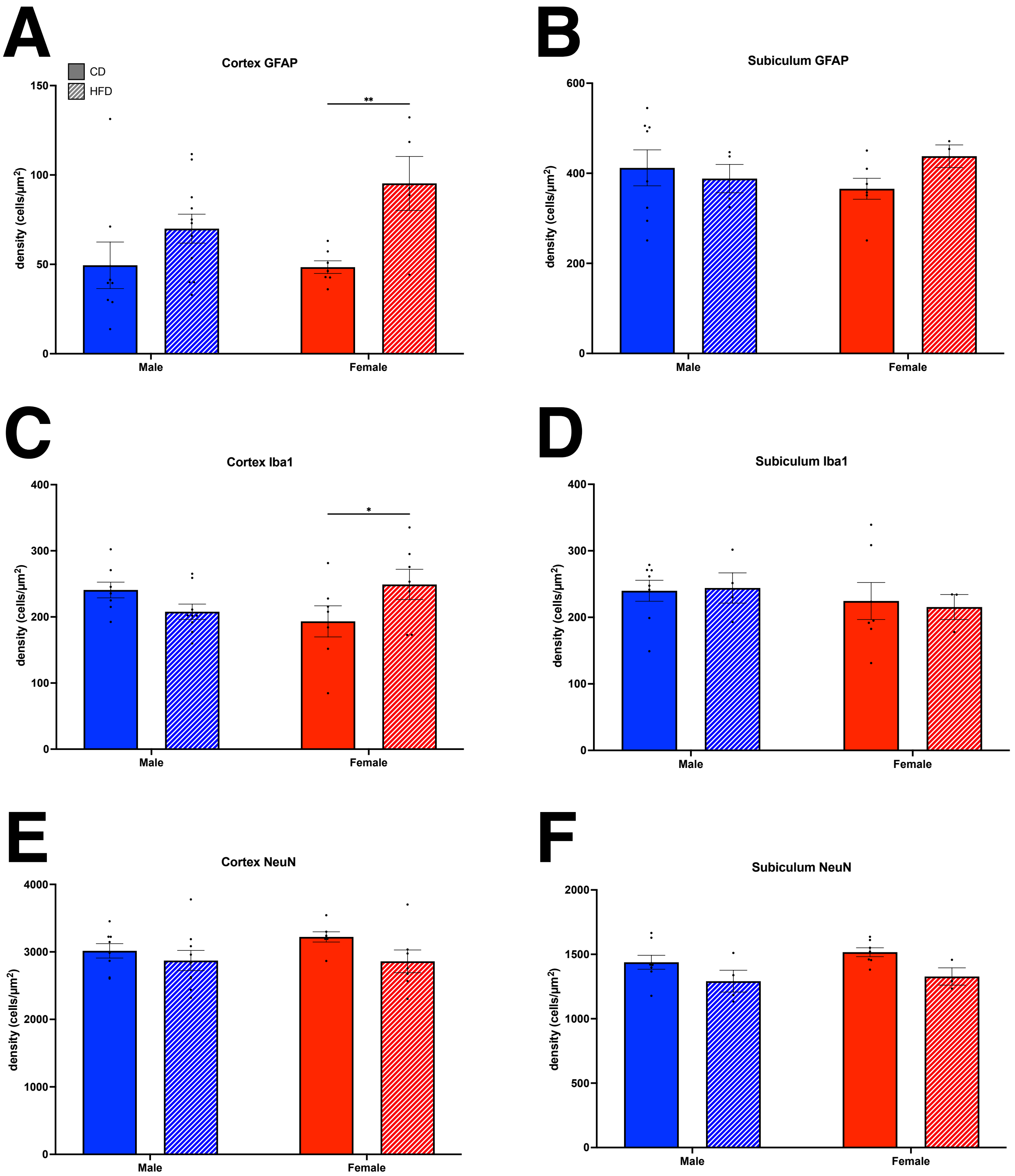

bioRxiv preprint doi: https://doi.org/10.1101/2023.12.19.571985; this version posted December 20, 2023. The copyright holder for this preprint (which was not certified by peer review) is the author/funder, who has granted bioRxiv a license to display the preprint in perpetuity. It is made available under aCC-BY 4.0 International license.

SUPPLEMENTAL FIGURE 6

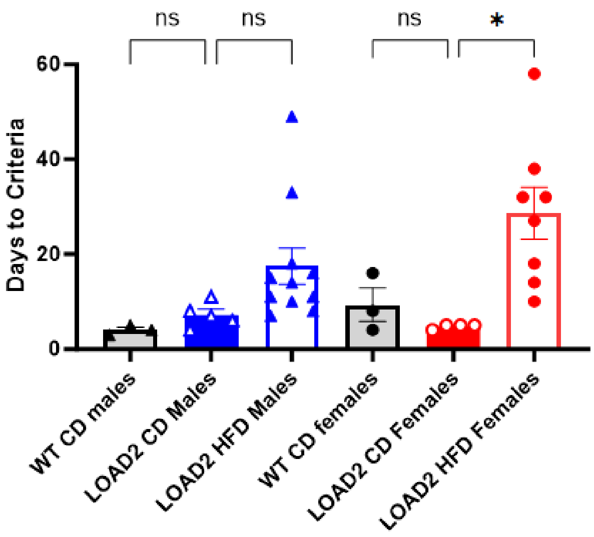

bioRxiv preprint doi: https://doi.org/10.1101/2023.12.19.571985; this version posted December 20, 2023. The copyright holder for this preprint (which was not certified by peer review) is the author/funder, who has granted bioRxiv a license to display the preprint in perpetuity. It is made available under aCC-BY 4.0 International license.

SUPPLEMENTAL FIGURE 7

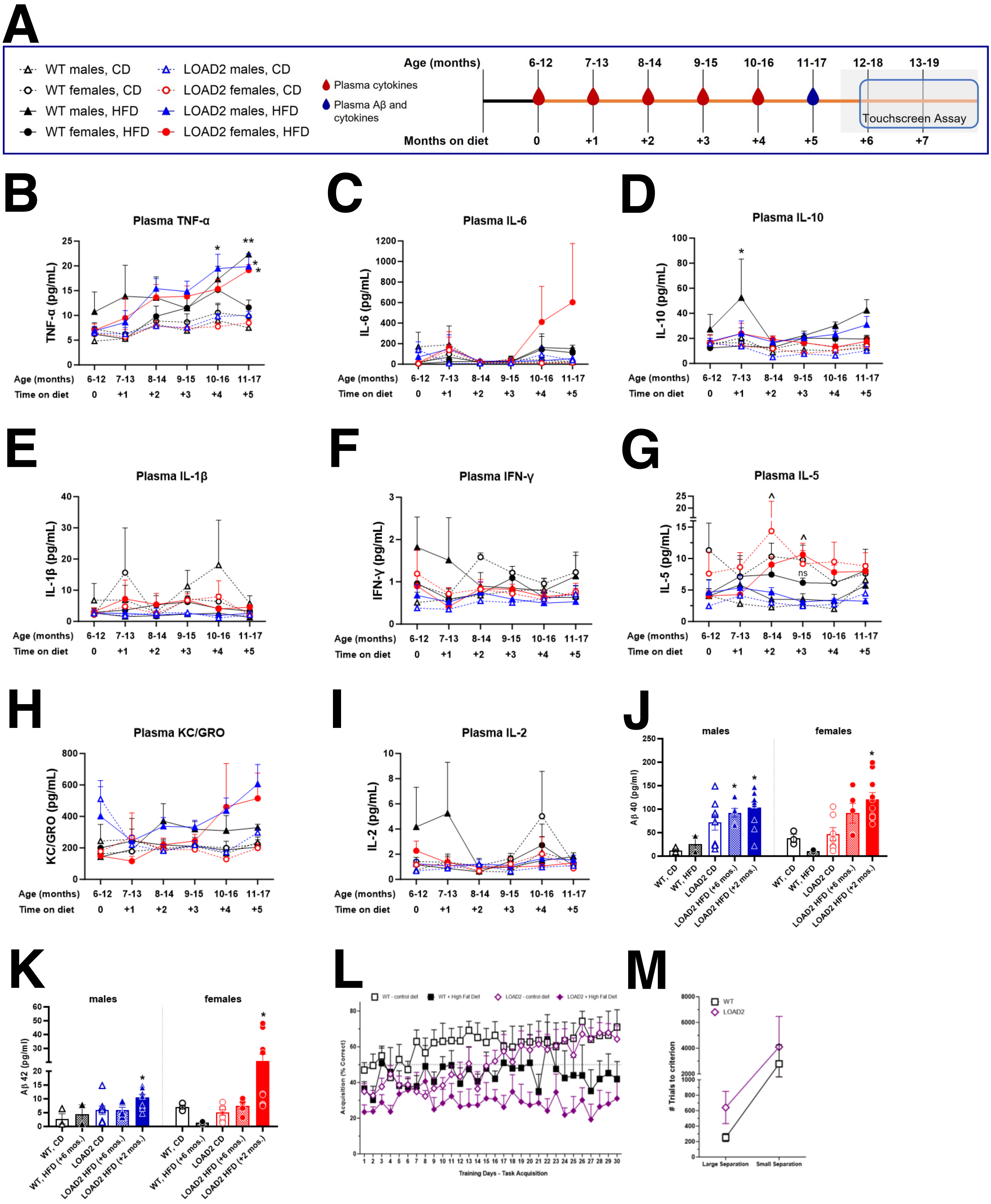

Supplement: Supplement 1 — SUPPLEMENTAL FIGURE 1: Study design. This was a multi-site, multi-cohort study. At each site, mice were provided a high fat diet or control diet starting at 2 months of age. At the Jackson Laboratory, the 18-month cohort only received a control diet. In cohort 1, four cohorts of mice were aged to 4, 12, 18, 24 months of age. Multiple ‘omics studies, behavior and cognition and neuropathology studies were completed. In cohort 2, at Indiana University, one cohort of mice were age to 18 months and plasma was collected at 4, 12 and 18 months for biomarker analysis. At each timepoint, mice had MRIs completed for volumetric analysis. Brains were collected at the termination of the study for neurodegeneration and neuroinflammation studies. In cohort 3 at Indiana University, two cohorts of mice were aged to 12 and 18 months and underwent PET/CT studies for two tracers, FDG and PTSM. In cohort 4 at University of Pittsburgh, mice were aged to 18 months, undergoing serial blood draws monthly and completing touch screen testing. Supplemental Figure 2: Longitudinal behavioral phenotyping of mice on high-fat diet. Males and females, of LOAD1 and LOAD2 genotypes, fed either control diet (CD) or high-fat diet (HFD) beginning at 2-months of age until 18-months of age were subjected to open field assay, measuring animal movements by way of total distance traveled (A) and total vertical activity (B) during 60 minute observation testing period. Rotarod assay measured the latency to fall times, over three consecutive trials, as a measure of motor coordination (C). (Three-way ANOVA [sex, genotype, diet effects]; *=p<0.05) Supplemental Figure 3: Immunohistochemistry analysis. Representative, collated images of whole hemisphere, coronal sections displaying both cortical and hippocampal regions of interest, used for glia cell density measurements and neuropathological assessment of brain tissue from cohort 1 (JAX). Brains are from 12mo females from either LOAD2 (control diet) and B6J.APPSAA [file NIHPP2023.12.19.571985v1-supplement-1.pdf]
